# Quantifying crime associated with drug use among a large cohort of sanctioned offenders in England and Wales

**DOI:** 10.1016/j.drugalcdep.2015.08.018

**Published:** 2015-10-01

**Authors:** Matthias Pierce, Karen Hayhurst, Sheila M. Bird, Matthew Hickman, Toby Seddon, Graham Dunn, Tim Millar

**Affiliations:** a Centre for Mental Health and Risk, University of Manchester, 4th Floor, Ellen Wilkinson Building, Oxford Road, M13 9PL Manchester, UK; b MRC Biostatistics Unit, Institute of Public Health, University Forvie Site, Robinson Way, CB2 0SR Cambridge, UK; c School of Social and Community Medicine, University of Bristol, Canynge Hall, 39 Whatley Road, BS8 2PS Bristol, UK; d School of Law, University of Manchester, 4.46A Williamson Building, Oxford Road, M13 9PL Manchester, UK; e Centre for Biostatistics, University of Manchester, Jean McFarlane Building (First Floor), Oxford Road, Manchester M13 9PL, UK

**Keywords:** Drug use and crime, Gender, Opioid and cocaine use, Acquisitive offending

## Abstract

**Aim:**

To assess the relationship between testing positive for opiates and/or cocaine and prior offending.

**Methods:**

139,925 persons (107,573 men) identified from a saliva test for opiate and cocaine metabolites following arrest in England and Wales, 1 April 2005–31 March 2009, were case-linked with 2-year recorded offending history. The prior offending rate, accounting for estimated incarceration periods, was calculated by: drug-test outcome; gender; four main crime categories (acquisitive, non-acquisitive, serious acquisitive, and non-serious acquisitive) and 16 sub-categories. Rate ratio (RR) compared opiate and/or cocaine positive to dual-negative testers. Adjusted rate ratio (aRR) controlled for age at drug test.

**Results:**

The relationship between testing positive for opiates and cocaine and prior 2-year offending was greater for women than men (aRR men 1.77; 95% CI: 1.75–1.79: women 3.51; 3.45–3.58). The association was weaker for those testing positive for opiates only (aRR: men: 1.66, 1.64–1.68; women 2.73, 2.66–2.80). Men testing positive for cocaine only had a lower rate of prior offending (aRR: 0.93, 0.92–0.94), women had a higher rate (aRR: 1.69, 1.64–1.74). The strongest associations were for non-serious acquisitive crimes (e.g. dually-positive: prostitution (women-only): aRR 24.9, 20.9–29.7; shoplifting: aRR men 4.05, 3.95–4.16; women 6.16, 5.92–6.41). Testing positive for opiates and cocaine was associated with violent offences among women (aRR: 1.54, 1.40–1.69) but not men (aRR: 0.98, 0.93–1.02).

**Conclusions:**

Among drug-tested offenders, opiate use is associated with elevated prior offending and the association is stronger for women than men. Cocaine use is associated with prior offending only among women.

## Highlights

• Opiate use is associated with elevated (acquisitive and non-acquisitive) offending.
• The association between drug use and crime is stronger for women than men.
• Cocaine use is associated with offending among males but not among females.
• Opiate and cocaine use is associated with 25 times the rate of prostitution (females only).

## 1 Introduction

Although the nature of the drugs–crime link is likely to be complex and multi-factorial (Seddon, 2000), it is well documented that those dependent on illicit substances are responsible for a disproportionate number of crimes, particularly crimes committed for financial gain (acquisitive crimes; Bennett et al., 2008, Parker et al., 1988). Involvement in income-generating crime may, to an extent, reflect users’ need to obtain funds to support their drug use (White and Gorman, 2000). Consistent with this, the association holds for those who are opiate or crack cocaine dependent and appears strongest for those who are dependent on both (Bennett et al., 2008). There is also support for an association with the use of other drugs, such as powder cocaine (Bennett and Holloway, 2007) or amphetamines (Klee and Morris, 1994).

The relationship is most evident for petty acquisitive crimes, such as shoplifting (Bennett et al., 2008). However, the extent to which drug dependence is associated with more serious acquisitive crime, such as robbery, or with non-acquisitive crime, such as violent offences, is less apparent (Parker and Auerhahn, 1998, White and Gorman, 2000). In England and Wales, initiatives to reduce drug-related crime have focussed, almost exclusively, on opiate and crack/cocaine users (Home Office, 2010, Home Office, 2011a), to whom a considerable proportion of all acquisitive crime has been attributed (MacDonald et al., 2005). Additionally, the prevalence of opiate and crack cocaine use, in particular, may influence trends in national crime rates (Morgan, 2014).

Much of the quantitative evidence for the drugs–crime link derives from interviews conducted with arrestees (Bennett et al., 2008). However, such information will be subject to recall and non-response bias. Record-linkage studies utilising criminal record data avoid these problems, but have typically relied on drug treatment cohorts and lacked a non-drug-using control group. The absence of a comparator group limits the inferences which can be made. For example, recent investigations of opioid treatment cohorts suggest higher rates of offending by men than women (Bukten et al., 2011, Degenhardt et al., 2013); but, as this finding is also observed in the general population (Ministry of Justice, 2012), it does not enlighten us about gender differences in the extent of the drugs–crime association. Two studies, identified in a recent meta-analysis (Bennett et al., 2008), which did compare men and women drug users with controls suggested that the drugs–crime link is stronger for women (Farabee et al., 2001, French et al., 2000). The meta-analysis concluded that: “more studies … are needed in order to test the relationship [between gender and the drugs-crime link] more thoroughly”.

Studies which have a (non-drug-using) control group rarely consider potential confounders of the drugs–crime relationship. In particular, age is a known important influence on offending rates, both for non-drug users (Farrington, 1986) and drug users (Horyniak et al., 2014). Thus, comparisons which fail to account for age may reflect differences in age composition, rather than differences related to drug use. Additionally, the existing literature rarely incorporates information about incarceration in the calculation of crime rates: crime rate estimates which fail to take account of periods of incarceration will be lower than rates based on ‘time in the community’ (Ferrante et al., 2009, Sutherland, 2013).

The current study seeks to quantify the relationship between opiate and/or cocaine use and 2-year prior historical offending by drawing on a large record-linkage cohort of 139,925 offenders who were drug tested and sanctioned following arrest. The prior conviction histories of criminally-active users were compared with criminally-active non-users over four main categories of crime (acquisitive, non-acquisitive, serious acquisitive, and non-serious acquisitive) and 16 sub-categories of crime. The association between testing positive and prior offending history was explored, separately by gender to gain insight on differences between men and women. Comparisons also account for differences in age and incarceration time.

The testable hypotheses are: (1) offenders testing positive for opiates or cocaine have a higher rate of prior past offending than negative testers; (2) those testing positive for both opiates and cocaine have the highest rate of prior offending; (3) the association between opiate/cocaine use among offenders and prior historical offending is stronger for women. The analysis also explores (4) whether the association is consistent over different categories of offence.

## 2 Methods

### 2.1 Data

The cohort comprised individuals recorded as receiving a salivary drug test following arrest in England and Wales (1 April 2005–31 March 2009). Two-year offending histories were extracted from linked Police National Computer (PNC) records.

The policy of drug testing was introduced to identify drug users in the criminal justice system and increase drug treatment participation (NTA, 2011). The policy operates in most large urban areas in England and Wales and involves a mandatory saliva test for opiate and cocaine (crack or powder form) metabolites following arrest for a ‘trigger’ offence (pre-defined as associated with problem drug use), or at the discretion of the police officer in charge of the custody area. Trigger offences are: theft; robbery; burglary; vehicle theft; supply or possession of cocaine or heroin (Home Office, 2011a). The Drug Test Record (DTR) records positive and negative saliva test results, test dates, reason for test and basic demographic information. Those who test positive are required to attend an initial assessment with a drugs worker who will help the user seek treatment and other support.

The PNC is an operational database containing information on all arrests resulting in a criminal charge. It records: type of offence; whether the charge resulted in a conviction, caution, warning or reprimand; court and sentencing outcomes; and offence date.

Subjects identified via the DTR were case-linked to PNC records for all offences occurring up to 31 March 2009 which resulted in a sanction (i.e., court conviction, police caution, warning or reprimand). Linkage was via the ‘minimal identifier’ derived from initials, date of birth and gender. These data were irreversibly encrypted prior to their release by source organisations, rendering them anonymous to the research team. The PNC records a unique personal identifier; multiple instances of this unique identifier paired with a single minimal identifier were taken as evidence that the minimal identifier was shared by more than one offender; these cases were removed.

The DTR cohort was selected via the first drug test satisfying the following criteria: (1) person tested aged 18–64; (2) completed test with an undisputed result; (3) subsequent charge and sanction. The latter criterion ensured that the analysis was based on established offenders so that it was not biased by unproven offences or poor linkage.

### 2.2 Outcome

Analysis considered those offences recorded as occurring during the 2 years prior to the drug test. Offences during the 2-week period immediately prior to the drug test were excluded. Thus, results were not unduly influenced by trigger offences prompting test administration.

Offences were classified into 16 UK Home Office categories (Home Office, 2011b), including sub-categories of ‘theft’, and additional categories of ‘breach’ and ‘prostitution’ (women only), both of which are prevalent amongst opioid and crack users (Booth et al., 2000, Gossop et al., 1994, Millar et al., 2008). Among women, there were few sexual offences (*n* = 72) and for women only this category was combined with ‘other indictable offences’.

Following a nationally-used indicator (Audit Commission, 2010), ‘serious acquisitive’ crimes were defined as: burglary, robbery, vehicle theft and theft from a vehicle. Non-serious acquisitive crime comprised the remainder of crime categories that confer financial gain (including prostitution and drug supply offences). Non-acquisitive crimes excluded drug misuse offences, for which higher rates among the DTR-positive subgroups were expected. Details of crime categories are provided in Supplementary Material A.

PNC records sentencing information and, in adherence to existing methodology (Sutherland, 2013), it was assumed that multiple prison sentences awarded at the same court appearance ran concurrently, unless stated otherwise. The estimated incarceration period was taken as half of the total sentence, as per the sentencing guidelines (HM Prison Services, 2008).

### 2.3 Analysis

The rate of offences per year was calculated separately for men and women, according to the offence category and drug test result. Follow-up time was calculated as 2 years per person minus estimated incarceration time. The rate ratio (RR), comparing those with a positive test to those who tested negative for both opiates and cocaine, was estimated using a Poisson regression model, with count of offences as the dependent variable. Adjustment for incarceration was made by including an ‘offset term’. The adjusted model included the categorical variable ‘age at drug test’ (18–19, 20–24, 25–29, 30–39, 40–49, 50–64 years). Due to the large cohort size and relative frequency of sanctioned offences, estimates were very precise and confidence intervals (CIs) often within 0.01 of the estimate so that CIs are reported in the text only where the level of statistical uncertainty warrants their inclusion.

## 3 Results

### 3.1 Description of the DTR cohort (Table 1)

**Fig. 1:**
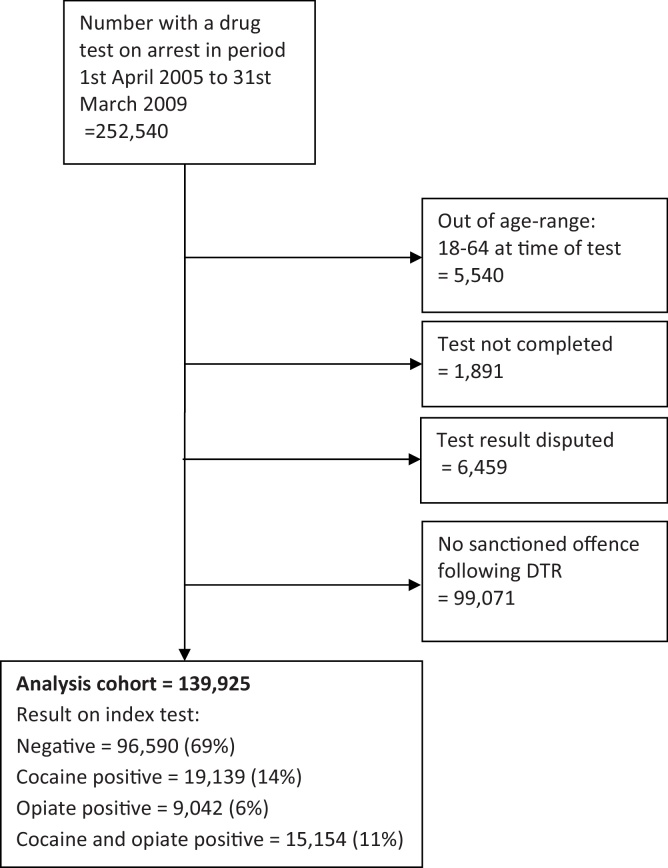
Construction of analysis cohort.

The DTR cohort consisted of 139,925 sanctioned offenders. Sixty-nine percent (97k/140k) tested negative both for opiates and for cocaine (hereafter “dually-negative”); 14% (19k) positive for cocaine only; 6% (9k) for opiates only and 11% (15k) positive both for opiates and for cocaine (hereafter “dually-positive”). A further 99,071 individuals had a drug test on arrest but no associated PNC record for an index charge, indicating that their index charge did not result in a recorded sanction or that linkage was not successful (Fig. 1).

The majority (77%, 108k/140k) were men, increasing to 86% (16k/19k) among those testing positive for cocaine only. Those testing positive for opiates tended to be older than those testing negative for opiates, regardless of their cocaine test result (mean age (SE): dually negative 29.3 (0.03); cocaine-only 29.4 (0.06); opiate-only 32.6 (0.09); dually positive 32.9 years (0.06)). Most (78%, 102k/140k) were of white ethnicity. Non-white subjects were less likely to test positive for opiates or cocaine (24% (7k/29k) vs. 33% (34k/102k)).

The cohort committed 364,845 recorded, sanctioned, crimes in the 2 years (minus 2 weeks) prior to their drug test, a rate of 1.30 per person year. Restricting follow-up to time in the community (i.e., subtracting estimated incarceration periods) increased the offending rate by 3.2%, to 1.35 per person year (Supplementary Material B). This increase was highest in men testing dually-positive (7.8%, from 2.01 to 2.16) and lowest in women testing dually-negative (0.4%, from 0.62 to 0.63) reflecting differences in average estimated incarceration time among subgroups (see Table 1).

### 3.2 Two year offending history by DTR results: main offending categories (Table 2)

The rate of 2-year prior (sanctioned) offending was greater among those testing positive for opiates (with or without a positive cocaine test) than for gender-matched dually-negative cases (age-adjusted rate ratio (aRR): opiate-only men 1.7; dually-positive men 1.8; opiate-only women 2.7; dually-positive women 3.5; Table 2). This association held for all main offending categories. After adjusting for age, men testing positive for cocaine only had a slightly lower rate of prior offending than those testing dually-negative (aRR: 0.9). In contrast, women testing positive for cocaine only had a higher rate of prior offending than dually-negative women (aRR: 1.7).

Men and women who tested dually-positive had a higher rate of prior offending than their gender-matched counterparts who tested positive for one or neither drug. This held for all main offence categories, with the exception of non-acquisitive crime, where men who tested dually-positive had a similar rate to those who tested positive for opiates only (aRR vs. negative testers 1.5 for both). Among dually-positive cases, prior offending rates for men and women were similar (2.16 and 2.22 offences per year, respectively). However, dually-positive men had a higher rate of prior serious acquisitive offences than dually-positive women (0.21 vs. 0.05) and a lower rate of non-serious acquisitive offences (0.75 vs. 1.10).

For all main categories of offences, the relative (multiplicative) increase in prior offending rate associated with testing positive for opiates was greater for women than for men. For example, the age-adjusted rate ratio for acquisitive offences associated with testing dually-positive was 3.2 for women and 2.2 for men. The absolute (additive) increase was also greater for women than for men for all main categories of offences with the exception of serious acquisitive crime where the rate difference for dually-positive compared to dually-negative cases was 0.08 crimes per year for men (from 0.13 to 0.21) and 0.03 crimes per year for women (from 0.02 to 0.05).

### 3.3 Offending subcategories: serious acquisitive crimes (Table 3)

Burglary was the most common serious acquisitive crime: 14% of dually-positive men had a prior sanctioned offence. The strongest associations were observed for theft from vehicle offences: the rate for dually-positive men was more than three times, and for dually-positive women more than five times, that for their dually-negative counterparts (aRR men 3.1; women 5.3).

Dually-positive and opiate positive only men had a lower unadjusted rate for robbery and theft of vehicle offences than dually-negative (RR dually-positive: 0.7 and 0.7; RR opiate-only: 0.6 and 0.7, respectively). However, the direction of association changed with age adjustment (aRR dually-positive: 1.4 and 1.5; opiate-only: 1.2 and 1.4, respectively). Among women, testing positive for opiates or cocaine was associated with a higher prior offending rate for all subcategories of serious acquisitive crime, albeit with a degree of uncertainty for robbery (aRR cocaine-only 1.3 95% CI 0.8–2.3; opiate-only 1.3 95% CI 0.6–2.5).

### 3.4 Offending subcategories: non-serious acquisitive crimes (Table 4)

Irrespective of age adjustment, for the vast majority of non-serious acquisitive crime sub-categories, men and women who tested positive for opiates (with or without a positive test for cocaine) had a higher rate of prior offending than those who tested dually-negative. One exception was for fraud and forgery offences where, among men, a positive test for opiates (regardless of cocaine test result) was associated with a lower offending rate (aRR dually-positive 0.7; opiate-only 0.7).

The strongest association was observed for prostitution (women only), especially among dually-positive testers, for whom the age-adjusted rate was almost 25 times higher than for negative testers (aRR 24.9). The next strongest association was observed for shoplifting (aRR men: opiate-only 3.5; dually-positive 4.1; women: opiate-only 4.7; dually-positive 6.2). Dually-positive women had a higher rate of prior sanctioned shoplifting offences per year than their male counterparts (0.70 vs. 0.50).

### 3.5 Offending subcategories: non acquisitive crimes (Table 5)

Within the non-acquisitive crime category, which comprises a heterogeneous group of offences, 16% of men were convicted of a violent offence in the 2 years prior to their drug test; 0.8% of sexual offences. For men, age-adjusted analysis revealed no clear association between testing positive and violent offences (i.e., dual positive aRR: 1.0). Among men, there was a negative association between testing positive and both prior sexual (aRR: cocaine-only 0.6, opiate-only 0.4, dually-positive 0.5) and criminal damage offences (aRR: cocaine-only 0.8, opiate-only 0.9, dually-positive 0.8).

In contrast to men, testing positive for either drug was associated with a higher rate of prior violent offending among women, with the strongest association among opiate-only positives (aRR: 1.9).

## 4 Discussion

This study confirms that sanctioned offenders who test positive for opiates have prior offending rates considerably higher than those who test dually-negative. This was evident both for men and women, and following adjustment for age and estimated incarceration periods. Counter to the initial hypothesis, men who tested positive for cocaine only did not have a higher rate of offending than those dually-negative; although the reverse was observed for women. Consistent with the second hypothesis, testing positive for both opiates and cocaine was associated with the highest prior offending rates.

There was strong support for the hypothesis that the association between drug use and prior offending is stronger for women than men. Adjusting for age differences, women who tested positive for both opiates and cocaine committed offences at a rate 3.5 times that for those who tested dually-negative; the figure for men was 1.8 times higher. Key to this observation is that, for those testing dually-negative, women had a much lower prior offending rate than men (0.63 vs. 1.38 per year), as has been observed elsewhere in the criminal justice system (Ministry of Justice Statistics Bulletin, 2013) and the general population (Ministry of Justice, 2012). Whilst the overall offending rate was similar for dually-positive men and women (2.0 vs. 2.2), the rate for non-serious acquisitive offences was higher for women than men (1.1 vs. 0.8), with the reverse observed for serious acquisitive offences (0.05 vs. 0.2). This indicates that gender differences in offending (Heidensohn and Silvestri, 2012) are less clear for drug users identified within the criminal justice system.

There was considerable variation in the strength of this association according to type of offence, as well as by gender and test result. For men, the association was stronger for acquisitive offences than non-acquisitive offences. For women, this hierarchy was less clear, with the strength of the association generally consistent across main offence categories. The strongest relationships with specific crime types were observed among those positive for both cocaine and opiates for prostitution (women only aRR = 24.9) and shoplifting (men aRR = 4.1, women aRR = 6.2).

Other important findings relate to violent and sexual offences. Previous studies indicate no clear link between drug use and violent offending (Parker and Auerhahn, 1998, White and Gorman, 2000). The findings from the current study confirm the lack of an association for men but suggest a positive association for women, although such offences were observed in just 8% (2.6k/32k) of women. Post hoc analysis highlighted that, among those dually-negative, in 31% (5715/18,171) of instances where a violent offence was committed, another offence also took place. This increased to 44% (1089/2491) among those dually-positive and indicates that prosecution for violent offences is associated with a wider offending pattern among drug users.

For prior fraud and forgery offences there was a negative association with testing positive for either drug among men and a negative association for cocaine-positive women. Previous UK research (McKeganey et al., 2006) has indicated high levels of self-reported fraud/forgery offences (Neale, 2004), but lacked the comparison group necessary to ascertain the extent of fraud and forgery offending among non-drug users; a characteristic design flaw in the majority of studies in this research area. Earlier work found little evidence of an association between fraud and opioid use (Hammersley et al., 1989).

The UK drugs–crime debate has been highly informed by the New English and Welsh Arrestee Drug Abuse Monitoring (NEW-ADAM) study (Bennett and Holloway, 2007). This study contrasted offending and drug use among 4645 interviewed arrestees. Their conclusion of a strong association between acquisitive offending and opiate use (odds ratio for heroin vs. no drug used (OR): 5.4, 95% CI 4.5–6.5) is supported here. However, NEW-ADAM did not contrast volume of offending (utilising instead a binary indicator), did not explore the association for specific offence types, and did not adjust for age, gender or incarceration. Thus, the current study provides greater and more specific insight on the nature of the drugs–crime link. A key difference relates to findings with respect to cocaine; the current study found no association between testing positive for cocaine and offending, whereas NEW-ADAM did observe an association with acquisitive offending (OR: 3.2, 95% CI: 2.6–3.8). However, NEW-ADAM excluded many subjects from those initially attending the sampled arrest suits and therefore eligible for interview (56% of eligible women, 45% of men), the majority due to lack of time in the custody suite. Additionally they recruited from all arrestees, rather than sanctioned offenders, as considered here.

The study had several strengths and limitations. The analysis of historical offending records limits the ability to attribute the findings to drug use. However the associations observed here will likely be applicable to future offending because of the high correlation between past and future offending. The cohort was selected on the basis of recorded offending, but only half of opiate, crack and cocaine users attending drug treatment report prior acquisitive offending (Hayhurst et al., 2012). Therefore the offending rates calculated here are considerably higher than would be expected in the population of drug users and non-users. The study findings are generalisable to the population subgroup of convicted offenders. This group is of particular policy interest because it is accessible for intervention.

Furthermore, selection was based on a completed drug test which was automatically prompted in the majority (93%) of the cohort by a ‘trigger’ offence. Although the effect of this selection was mitigated by excluding the 2 weeks prior to the drug test, this may skew the cohort towards those who commit these types of offences. Again, this is a group of particular policy interest because theft offences form the largest offending subgroup. These selection effects may function differently for drug positive and drug negative offenders. Differently-designed work would be needed to establish whether the associations observed in this drug-test sanctioned cohort would apply prospectively or to the wider population.

A short 2-year historical period was selected to increase the likelihood that drug test results are relevant to the observation period. However, some misclassification may remain. The saliva test has an estimated sensitivity of 81.5% and specificity of 99.3% for opiates (Kacinko et al., 2004) and 89.4% and 92.2%, for cocaine (Kolbrich et al., 2003). Additionally, a negative drug test may reflect non-use immediately prior to arrest rather than at the time of the offence. Such misclassification will result in an underestimation of the true differences between groups.

The test for cocaine does not differentiate between crack and powder cocaine. Previous studies indicate that powder cocaine users commit fewer acquisitive offences than crack cocaine users (Gossop et al., 2002, Gossop et al., 2006). The results obtained in the current study may be due to gender differences in the prevalence of crack versus powder cocaine use among arrestees.

Sanctioned offending significantly underestimates the true rate of offending and, for the findings to be accurate, underestimation must apply equally to each subgroup. Others have highlighted (Stevens, 2008) that progression through the criminal justice system from arrest to sanction involves police discretion and such discretion may not be applied equally to drug users and non-drug users. For example, drug-using offenders may be more likely than non-using offenders to be apprehended or charged (Bond and Sheridan, 2007, Stevens, 2008).

As far as we are aware, this is the first record-linkage study to investigate associations between drug use and criminal offending using a comparison group and carrying out adjustments both for age and incarceration periods. A key strength of the study is its large cohort, linked to an objective biological sample, providing the necessary power to examine in detail associations between opiate and/or cocaine use and offending for specific offence categories, separately by gender. In total there were 32 separate analyses: one for each of the offending subcategories (*n* = 16) and gender combination. If these were conservatively assumed to be independent, and using the Bonferonni correction for statistical significance (0.05/32 = 0.002), all global tests remained significant at this level. The extent of differences observed between men and women highlights the necessity of accounting for gender in this type of study. Additionally, these findings highlight the importance of adjusting for age and, to a lesser extent, periods of incarceration. For men in particular, the relationship between testing positive for opiates and serious acquisitive offending was only apparent after age adjustment, indicating strong confounding by age. Adjusting for potential confounders unavailable in the current study, i.e., socio-economic status or educational attainment, may further refine the relationships observed here.

This study strengthens the evidence of an association between opiate use and prior offending, by showing that it is independent of age, incarceration and gender. The study also provides greater nuance into the drugs–crime link than previously available and shows how the strength of association varies according to gender and the category of offence under consideration. The findings of this study should inform future discussion of the relationship between drugs and crime. An extension to this work involves examining rates of offending before and after the onset of opiate use to clarify the longitudinal relationship between drug-use initiation and offending. Another extension would be to link the cohort to treatment records and analyse the data prospectively, identifying the offending differences between non-drug users, drug users who were referred to treatment and those who were not.

The study has important implications. First, gender clearly influences the extent and nature of the association between drug use and prior offending. Thus, targeted, gender-specific interventions may maximise success. Additionally, the most common offences committed by opiate users were non-serious acquisitive offences, particularly shoplifting, which, in addition to prostitution amongst women (not currently included in the list of ‘trigger’ offences which prompt drug testing), had the strongest association with opiate use.

The impact of interventions within the criminal justice system, such the UK's Drug Intervention Programme (DIP), may be most evident in respect of these types of crime. Finally, insofar as it was not a predictor of offending, cocaine testing for men may not be a cost-effective method of identification.

## Role of the funding source

This research was funded as part of the Insights study by the UK Medical Research Council (MR/J013560/1). Sheila Bird is funded by Medical Research Council programme number MC_U105260794. The MRC had no further role in study design; in the collection, analysis and interpretation of data; in the writing of the report; or in the decision to submit the paper for publication. The Home Office has been provided with a pre-submission version of this manuscript but has not exerted any editorial control over, or commented on, its content.

## Contributors

Millar, Pierce and Hayhurst conceived of the study. Pierce with input from Bird wrote the analysis plan. Pierce analysed the data and wrote a first draft of the manuscript. Millar, Bird and Dunn supervised data analysis. All interpreted the data and edited the manuscript.

## Conflict of interest

Millar has received research funding from the UK National Treatment Agency for Substance Misuse and the Home Office. He is a member of the organising committee for, and chairs, conferences supported by unrestricted educational grants from Reckitt Benckiser, Lundbeck, Martindale Pharma, and Britannia Pharmaceuticals Ltd, for which he receives no personal remuneration. He is a member of the Advisory Council on the Misuse of Drugs. Bird holds GSK shares. She is an MRC programme leader. She chaired Home Office's Surveys, Design and Statistics Subcommittee (SDSSC) when SDSSC published its report on 21st Century Drugs and Statistical Science. She has previously served as UK representative on the Scientific Committee for European Monitoring Centre for Drugs and Drug Addiction. She is co-principal investigator for MRC-funded, prison-based N-ALIVE pilot Trial. Seddon has received research funding from the UK National Treatment Agency for Substance Misuse and the Home Office.

## Figures and Tables

**Table 1:** Description of cohort.

	DTR result			
	Dually negative (*n* = 96,590)	Cocaine positive only (*n* = 19,139)	Opiate positive only (*n* = 9042)	Cocaine and opiate positive (*n* = 15,154)
Gender				
Men	72,643 (75)	16,430 (86)	7046 (78)	11,454 (76)
Women	23,947 (25)	2709 (14)	1996 (22)	3700 (24)
				
Mean age at test (±SD)	29.3 (±10.3)	29.4 (±8.8)	32.6 (±8.4)	32.9 (±7.9)
Ethnicity reported at index test				
White ethnic groups	68,232 (76)	15,078 (82)	7250 (87)	11,798 (83)
Non white ethnic groups	21,904 (24)	3293 (18)	1061 (13)	2380 (17)
Index test following arrest for ‘trigger’ offence				
Yes	89,755 (93)	17,499 (91)	8481 (94)	13,991 (92)
No	6835 (7)	1640 (9)	561 (6)	1163 (8)
Number of people with a crime over follow-up				
Men (% of all men)	43,076 (59)	8782 (53)	4794 (68)	7949 (69)
Women (% of all women)	9556 (40)	1336 (49)	1240 (62)	2562 (69)
Number of crimes				
Men	194,195	39,053	26,990	45,976
Women	30,145	5827	6715	15,944
Mean number of crimes (±SD)				
Men	2.67 (±4.38)	2.38 (±4.01)	3.83 (±5.14)	4.01 (±5.25)
Women	1.26 (±3.20)	2.15 (±4.05)	3.36 (±5.08)	4.31 (±5.77)
Person years of follow-up				
Men	145,286	32,860	14,092	22,908
Women	47,894	5418	3992	7400
Mean estimated incarceration time (years) over 2 year history (±SD)				
Men	0.125 (±0.265)	0.145 (±0.289)	0.181 (±0.317)	0.195 (±0.318)
Women	0.032 (±0.118)	0.074 (±0.182)	0.098 (±0.208)	0.110 (±0.192)

Numbers in round brackets are percentages, unless otherwise stated.

**Table 2:** Rate per year, rate ratio and age-adjusted rate ratio for the main offence categories, by drug test result and gender.

		Men, *n* = 107,573			Women, *n* = 32,352		
Offence category	DTR subgroup	Rate	RR	aRR	Rate	RR	aRR
All offences	Dually negative	1.378	Ref.	Ref.	0.632	Ref.	Ref.
Cocaine only	1.235	0.90 [0.89, 0.91]	0.93 [0.92, 0.94]	1.090	1.73 [1.68, 1.77]	1.69 [1.64, 1.74]
Opiate-only	2.047	1.49 [1.47, 1.50]	1.66 [1.64, 1.68]	1.720	2.72 [2.65, 2.79]	2.73 [2.66, 2.80]
Opiate and cocaine	2.164	1.57 [1.55, 1.59]	1.77 [1.75, 1.79]	2.221	3.51 [3.45, 3.58]	3.46 [3.39, 3.53]
Acquisitive offences	Dually negative	0.455	Ref.	Ref.	0.349	Ref.	Ref.
Cocaine-only	0.372	0.82 [0.80, 0.83]	0.83 [0.81, 0.85]	0.532	1.52 [1.46, 1.58]	1.49 [1.43, 1.55]
Opiate-only	0.853	1.87 [1.84, 1.91]	1.95 [1.91, 1.99]	0.876	2.51 [2.42, 2.60]	2.46 [2.37, 2.55]
Opiate and cocaine	0.962	2.11 [2.08, 2.15]	2.21 [2.17, 2.24]	1.161	3.32 [3.24, 3.41]	3.21 [3.13, 3.30]
Serious acquisitive offences	Dually negative	0.133	Ref.	Ref.	0.017	Ref.	Ref.
Cocaine-only	0.110	0.83 [0.80, 0.86]	0.92 [0.89, 0.95]	0.033	1.94 [1.65, 2.29]	2.05 [1.74, 2.41]
Opiate-only	0.179	1.35 [1.29, 1.41]	1.84 [1.76, 1.92]	0.039	2.31 [1.95, 2.75]	2.74 [2.30, 3.27]
Opiate and cocaine	0.208	1.57 [1.52, 1.62]	2.19 [2.11, 2.27]	0.051	3.02 [2.67, 3.41]	3.63 [3.19, 4.12]
Non-serious acquisitive offences	Dually negative	0.323	Ref.	Ref.	0.332	Ref.	Ref.
Cocaine-only	0.262	0.81 [0.79, 0.83]	0.80 [0.78, 0.82]	0.499	1.50 [1.44, 1.56]	1.46 [1.40, 1.52]
Opiate-only	0.674	2.09 [2.04, 2.14]	1.98 [1.94, 2.03]	0.836	2.52 [2.42, 2.61]	2.45 [2.36, 2.54]
Opiate and cocaine	0.754	2.34 [2.29, 2.38]	2.21 [2.17, 2.25]	1.109	3.34 [3.25, 3.43]	3.19 [3.11, 3.28]
Non acquisitive offences	Dually negative	0.856	Ref.	Ref.	0.268	Ref.	Ref.
Cocaine-only	0.780	0.91 [0.90, 0.92]	0.95 [0.94, 0.96]	0.517	1.93 [1.85, 2.01]	1.89 [1.82, 1.97]
Opiate-only	1.088	1.27 [1.25, 1.29]	1.47 [1.45, 1.50]	0.779	2.90 [2.79, 3.02]	2.99 [2.88, 3.12]
Opiate and cocaine	1.085	1.27 [1.25, 1.29]	1.49 [1.47, 1.51]	0.985	3.67 [3.56, 3.78]	3.71 [3.60, 3.82]

Rate = rate of offending per person year; RR = rate ratio; aRR = rate ratio adjusted for age (counts of offences and age-covariate estimates available in Appendix C).

**Table 3:** Serious acquisitive crime subcategories: offending rate, rate ratio and age-adjusted rate ratio, by drug test result and gender.

		Men, *n* = 107,573				Women, *n* = 32,352			
Offence category	DTR subgroup	%	Rate	RR	aRR	%	Rate	RR	aRR
Burglary	Dually negative	8.3	0.065	Ref.	Ref.	1.5	0.009	Ref.	Ref.
Cocaine-only	6.8	0.057	0.87 [0.83, 0.92]	0.92 [0.87, 0.97]	2.9	0.021	2.28 [1.85, 2.80]	2.34 [1.90, 2.88]
Opiate-only	13.5	0.109	1.67 [1.58, 1.77]	1.93 [1.82, 2.04]	3.7	0.028	3.09 [2.51, 3.80]	3.44 [2.78, 4.24]
Opiate and cocaine	14.3	0.128	1.97 [1.89, 2.06]	2.30 [2.20, 2.41]	5.2	0.036	3.92 [3.36, 4.57]	4.38 [3.74, 5.14]
Theft of vehicle	Dually negative	4.9	0.036	Ref.	Ref.	0.8	0.004	Ref.	Ref.
Cocaine-only	3.7	0.026	0.74 [0.68, 0.79]	0.90 [0.84, 0.97]	1.3	0.007	1.76 [1.25, 2.47]	1.89 [1.34, 2.66]
Opiate-only	3.7	0.025	0.70 [0.63, 0.78]	1.36 [1.21, 1.52]	1.0	0.006	1.44 [0.95, 2.21]	1.81 [1.18, 2.77]
Opiate and cocaine	3.5	0.026	0.74 [0.68, 0.81]	1.54 [1.40, 1.68]	1.2	0.007	1.73 [1.28, 2.35]	2.20 [1.61, 3.01]
Theft from vehicle	Dually negative	2.4	0.018	Ref.	Ref.	0.1	0.001	Ref.	Ref.
Cocaine-only	2.2	0.019	1.05 [0.96, 1.14]	1.10 [1.01, 1.21]	0.3	0.002	1.67 [0.82, 3.41]	1.81 [0.89, 3.71]
Opiate-only	4.0	0.037	2.07 [1.88, 2.28]	2.52 [2.28, 2.79]	0.4	0.003	2.55 [1.29, 5.03]	3.25 [1.63, 6.48]
Opiate and cocaine	4.4	0.044	2.47 [2.29, 2.66]	3.08 [2.84, 3.34]	0.7	0.004	4.01 [2.53, 6.37]	5.27 [3.24, 8.58]
Robbery	Dually negative	1.9	0.014	Ref.	Ref.	0.4	0.003	Ref.	Ref.
Cocaine-only	1.2	0.008	0.57 [0.50, 0.65]	0.71 [0.63, 0.81]	0.6	0.003	1.16 [0.69, 1.95]	1.34 [0.79, 2.25]
Opiate-only	1.1	0.008	0.59 [0.48, 0.71]	1.16 [0.96, 1.41]	0.3	0.002	0.89 [0.45, 1.76]	1.28 [0.64, 2.52]
Opiate and cocaine	1.3	0.009	0.65 [0.56, 0.75]	1.37 [1.17, 1.59]	0.7	0.004	1.51 [1.00, 2.28]	2.29 [1.50, 3.52]

% = percentage with crime over follow-up; rate = rate of offending per person year; RR = rate ratio; aRR = rate ratio adjusted for age (counts of offences and age-covariate estimates available in Appendix C).

**Table 4:** Non-serious acquisitive crime subcategories: offending rate, rate ratio and age-adjusted rate ratio, by drug test result and gender.

		Men, *n* = 107,573				Women, *n* = 32,352			
Offence category	Group	%	Rate	RR	aRR	%	Rate	RR	aRR
Shoplifting	Dually negative	9.3	0.111	Ref.	Ref.	10.6	0.110	Ref.	Ref.
Cocaine-only	8.3	0.114	1.03 [1.00, 1.07]	1.00 [0.96, 1.03]	19.0	0.246	2.25 [2.12, 2.39]	2.18 [2.05, 2.32]
Opiate-only	26.1	0.430	3.89 [3.78, 4.01]	3.49 [3.38, 3.60]	32.4	0.524	4.78 [4.54, 5.03]	4.71 [4.47, 4.96]
Opiate and cocaine	28.0	0.504	4.56 [4.45, 4.68]	4.05 [3.95, 4.16]	38.0	0.700	6.38 [6.14, 6.63]	6.16 [5.92, 6.41]
Other theft and handling	Dually negative	13.2	0.105	Ref.	Ref.	12.5	0.102	Ref.	Ref.
Cocaine-only	8.5	0.066	0.63 [0.60, 0.66]	0.64 [0.61, 0.67]	10.7	0.089	0.87 [0.79, 0.95]	0.86 [0.78, 0.95]
Opiate-only	14.7	0.118	1.12 [1.06, 1.18]	1.15 [1.09, 1.21]	15.2	0.128	1.25 [1.14, 1.37]	1.24 [1.13, 1.36]
Opiate and cocaine	15.0	0.123	1.17 [1.13, 1.22]	1.20 [1.15, 1.26]	15.1	0.140	1.37 [1.28, 1.46]	1.35 [1.26, 1.45]
Fraud and forgery	Dually negative	6.1	0.067	Ref.	Ref.	8.2	0.098	Ref.	Ref.
Cocaine-only	3.3	0.037	0.56 [0.53, 0.59]	0.53 [0.50, 0.56]	6.9	0.084	0.86 [0.78, 0.95]	0.83 [0.76, 0.92]
Opiate-only	4.9	0.056	0.85 [0.78, 0.91]	0.73 [0.68, 0.79]	9.0	0.108	1.10 [1.00, 1.22]	1.04 [0.94, 1.15]
Opiate and cocaine	4.8	0.055	0.83 [0.78, 0.88]	0.71 [0.67, 0.75]	8.4	0.104	1.07 [0.99, 1.15]	0.99 [0.91, 1.07]
Drug supply offences	Dually negative	2.6	0.032	Ref.	Ref.	1.0	0.009	Ref.	Ref.
Cocaine-only	3.2	0.038	1.20 [1.13, 1.28]	1.23 [1.15, 1.31]	2.1	0.021	2.19 [1.78, 2.69]	2.07 [1.68, 2.55]
Opiate-only	4.0	0.059	1.86 [1.72, 2.00]	2.04 [1.88, 2.20]	3.0	0.035	3.70 [3.05, 4.47]	3.51 [2.89, 4.25]
Opiate and cocaine	3.6	0.057	1.79 [1.68, 1.90]	1.97 [1.85, 2.11]	2.7	0.037	3.92 [3.37, 4.56]	3.56 [3.05, 4.16]
Theft from person	Dually negative	1.3	0.009	Ref.	Ref.	1.2	0.010	Ref.	Ref.
Cocaine-only	1.0	0.007	0.77 [0.66, 0.89]	0.81 [0.70, 0.94]	1.9	0.015	1.60 [1.26, 2.02]	1.62 [1.28, 2.06]
Opiate-only	1.6	0.011	1.25 [1.05, 1.48]	1.45 [1.22, 1.74]	1.9	0.014	1.44 [1.09, 1.91]	1.52 [1.15, 2.02]
Opiate and cocaine	1.8	0.015	1.68 [1.48, 1.90]	1.97 [1.73, 2.25]	3.5	0.032	3.37 [2.87, 3.94]	3.56 [3.02, 4.19]
Prostitution	Dually negative					0.3	0.003	Ref.	Ref.
Cocaine-only					3.2	0.043	13.0 [10.6, 15.9]	12.1 [9.90, 14.9]
Opiate-only					2.4	0.028	8.45 [6.63, 10.8]	7.54 [5.91, 9.63]
Opiate and cocaine					7.3	0.096	28.8 [24.2, 34.2]	24.9 [20.9, 29.7]

% = percentage with crime over follow-up; rate = rate of offending per person year; RR = rate ratio; aRR = rate ratio adjusted for age (counts of offences and age-covariate estimates available in Appendix C).

**Table 5:** Non-acquisitive crime subcategories: offending rate, rate ratio and age-adjusted rate ratio, by drug test result and gender.

		Men, *n* = 107,573				Women, *n* = 32,352			
Offence category	Statistic	%	Rate	RR	aRR	%	Rate	RR	aRR
Other summary offences	Dually negative	23.6	0.327	Ref.	Ref.	7.3	0.075	Ref.	Ref.
Cocaine-only	23.0	0.304	0.93 [0.91, 0.95]	0.95 [0.93, 0.98]	11.8	0.119	1.58 [1.45, 1.71]	1.54 [1.42, 1.68]
Opiate-only	23.9	0.363	1.11 [1.08, 1.14]	1.24 [1.20, 1.28]	13.4	0.137	1.81 [1.65, 1.98]	1.84 [1.68, 2.01]
Opiate and cocaine	24.0	0.356	1.09 [1.06, 1.11]	1.23 [1.20, 1.26]	13.8	0.162	2.15 [2.01, 2.29]	2.13 [1.99, 2.28]
Breach	Dually negative	20.8	0.260	Ref.	Ref.	8.2	0.103	Ref.	Ref.
Cocaine-only	21.1	0.249	0.96 [0.93, 0.98]	0.99 [0.97, 1.02]	20.6	0.275	2.67 [2.52, 2.83]	2.56 [2.42, 2.72]
Opiate-only	36.2	0.503	1.94 [1.89, 1.99]	2.22 [2.16, 2.28]	33.5	0.501	4.86 [4.61, 5.12]	4.85 [4.60, 5.11]
Opiate and cocaine	38.4	0.522	2.01 [1.96, 2.05]	2.33 [2.28, 2.38]	43.6	0.675	6.54 [6.29, 6.81]	6.32 [6.07, 6.59]
Violence against the person	Dually negative	16.7	0.127	Ref.	Ref.	7.1	0.055	Ref.	Ref.
Cocaine-only	15.4	0.115	0.90 [0.87, 0.93]	0.94 [0.91, 0.97]	10.0	0.074	1.35 [1.21, 1.50]	1.38 [1.24, 1.53]
Opiate-only	14.7	0.111	0.87 [0.83, 0.92]	1.01 [0.96, 1.07]	12.1	0.094	1.72 [1.54, 1.91]	1.92 [1.72, 2.14]
Opiate and cocaine	14.3	0.105	0.83 [0.79, 0.87]	0.98 [0.93, 1.02]	10.9	0.076	1.39 [1.27, 1.52]	1.54 [1.40, 1.69]
Criminal damage	Dually negative	10.8	0.082	Ref.	Ref.	3.2	0.023	Ref.	Ref.
Cocaine-only	9.0	0.062	0.76 [0.73, 0.80]	0.83 [0.79, 0.87]	3.6	0.023	0.99 [0.82, 1.20]	1.03 [0.85, 1.24]
Opiate-only	7.6	0.053	0.65 [0.60, 0.70]	0.87 [0.80, 0.94]	4.0	0.025	1.09 [0.89, 1.34]	1.24 [1.01, 1.53]
Opiate and cocaine	6.8	0.046	0.56 [0.52, 0.59]	0.76 [0.71, 0.81]	3.2	0.020	0.86 [0.72, 1.02]	0.98 [0.82, 1.17]
Other indictable offences	Dually negative	5.6	0.051	Ref.	Ref.	1.6	0.012	Ref.	Ref.
Cocaine-only	5.3	0.045	0.88 [0.83, 0.93]	1.00 [0.94, 1.06]	2.4	0.027	2.21 [1.84, 2.66]	2.20 [1.83, 2.65]
Opiate-only	5.8	0.053	1.04 [0.96, 1.12]	1.42 [1.32, 1.54]	2.8	0.022	1.83 [1.46, 2.30]	1.90 [1.51, 2.39]
Opiate and cocaine	5.7	0.051	1.00 [0.94, 1.07]	1.40 [1.31, 1.49]	3.9	0.052	4.34 [3.81, 4.94]	4.47 [3.91, 5.12]
Sexual	Dually negative	0.9	0.008	Ref.	Ref.				
Cocaine-only	0.6	0.005	0.62 [0.52, 0.73]	0.62 [0.53, 0.73]				
Opiate-only	0.5	0.004	0.47 [0.35, 0.62]	0.44 [0.33, 0.59]				
Opiate and cocaine	0.4	0.005	0.56 [0.46, 0.69]	0.52 [0.43, 0.65]				

% = percentage with crime over follow-up; rate = rate of offending per person year; RR = rate ratio; aRR = rate ratio adjusted for age (counts of offences and age-covariate estimates available in Appendix C).
