# Supplementary material for: Quantifying crime associated with drug use among a large cohort of sanctioned offenders in England and Wales
Source: Drug Alcohol Depend. 2015 Oct 1;155:52–9. doi: 10.1016/j.drugalcdep.2015.08.018 (PMC4768078; doi:10.1016/j.drugalcdep.2015.08.018)
Supplement: Supplementary file 1 [file mmc1.docx]

**Supplementary Material for the article**

**Quantifying crime associated with drug use among a large cohort of sanctioned offenders in England and Wales**

*Matthias Pierce^1^, Karen Hayhurst^1^, Sheila M Bird^2^, Matthew Hickman^3^, Toby Seddon^4^, Graham Dunn^5^, Tim Millar^1^*

*^1^Centre for Mental Health and Risk, University of Manchester,* *4th Floor, Ellen Wilkinson Building, Oxford Road, UK, M13 9PL* [*matthias.pierce@manchester.ac.uk*](mailto:matthias.pierce@manchester.ac.uk)*;* [*karen.hayhurst@manchester.ac.uk*](mailto:karen.hayhurst@manchester.ac.uk)*; tim.millar@manchester.ac.uk*

*^2^MRC Biostatistics Unit, Institute of Public Health, University Forvie Site, Robinson Way, Cambridge. UK, CB2 0SR sheila.bird@mrc-bsu.cam.ac.uk*

*^3^School of Social and Community Medicine, University of Bristol, Canynge Hall, 39 Whatley Road, Bristol, UK, BS8 2PS matthew.hickman@bristol.ac.uk*

*^4^School of Law, University of Manchester, 4.46A Williamson Building, Oxford Road, UK, M13 9PL toby.seddon@manchester.ac.uk*

*^5^ Centre for Biostatistics, University of Manchester, Jean McFarlane Building (First Floor), Oxford Road, UK, M13 9PL graham.dunn@manchester.ac.uk*

*Correspondence to: Tim Millar tim.millar@manchester.ac.uk*

**This material supplements but does not replace the peer-reviewed article in**

***Drug and Alcohol Dependence.***

**Supplementary Material A A Offence classification**

| Offence group | Details of offences |
| --- | --- |
| **Serious acquisitive offences** |  |
| Burglary | Burglary in a dwelling; Aggravated burglary in a dwelling; Burglary other than in a dwelling; Aggravated burglary in a building other than a dwelling; Going equipped for stealing, etc, |
| Robbery | Robbery and assaults with intent to rob; |
| Theft of a vehicle | Aggravated vehicle taking; Stealing and unauthorised taking of motor vehicle |
| Stealing from vehicles: | From motor vehicles; From other vehicles |
| **Non-serious acquisitive offences** |  |
| Theft from shop | Stealing from shops and stalls |
| Other theft and handling stolen goods | Money laundering offences (not drugs); Stealing by an employee; Abstracting electricity; Stealing pedal cycles; Stealing from automatic machines and meters; Other theft or unauthorised taking; Handling stolen goods |
| Fraud and forgery | Frauds by company directors, etc; False accounting; Other fraud Bankruptcy and insolvency; Forgery, etc. of prescription; Other forgery, etc; Blackmail; |
| Prostitution |  |
| Theft from person | Stealing from the person of another; |
| Drug offences – supply | Including possession with intent |
| **Non-acquisitive offences** |  |
| Violence against the person | Murder; Attempted murder; Threats, conspiracy or incitement to murder; Manslaughter, etc; Wounding and other act endangering life; Endangering railway passenger: Endangering life at sea; Cruelty to or neglect of children; Abandoning children under two years; Child abduction; Procuring illegal abortion; Concealment of birth; common assault |
| Sexual offences | Sexual assault on a male; Rape; Sexual assault on female; Sexual activity (male and female) (including with a child under 13); Sexual activity (male and female) (including with a child under 16); Familial sexual offences (incest); Exploitation of prostitution; Trafficking for sexual exploitation: Bigamy; Sexual activity etc. with a person with a mental disorder; Abuse of children through prostitution and pornography; Abuse of Trust - sexual offences; Gross indecency with children; Miscellaneous sexual offences |
| Criminal damage | Arson; Criminal damage endangering life; Other criminal damage; Threat and possession with intent to commit criminal damage; summary criminal damage offences |
| Other indictable offences | Breach of ASBO; Kidnapping; Riot; Violent disorder; Other offences against the State or Public Order; Perjury; Libel; Offender Management Offences; Betting, gaming and lotteries; Suicide; Immigration Acts; Perverting the course of justice; Absconding from lawful custody; Offences against the Firearms Act 1968 and other Firearms Acts; Offences against laws relating to Customs, Excise and Inland Revenue; Offences relating to bail; Trade Descriptions Act and similar legislation; Health and Safety at Work, etc. Act 1974; Obscene publications etc and protected sexual material; Protection from Eviction; Adulteration of food; Knives Act 1997 and other related offensive weapon Acts and Regulations not dealt with elsewhere; Public Health; Town and Country Planning Act 1990; Disclosure, obstruction, false or misleading statements etc; Other indictable offences; Dangerous driving; Driving licence related offences; Vehicle insurance offences; Vehicle registration and licence offences; Fraud, forgery, etc. associated with vehicle or driver records; Obstruction, waiting and parking offences; Miscellaneous indictable offences; |
| Summary offences | Animals; Explosives, Firearms etc; Financial, etc ; Game Laws ; Highways Acts; Public Order offences; Intoxicating Liquor Laws; Juvenile smoking; Labour Laws; Miscellaneous summary offences; Naval, Military, Air Force and Air Navigation Law; Offences against certain local regulations; Revenue Laws; Theft (summary offences); Vagrancy offences; Motoring offences; |
| Breach |  |

**Supplementary Material B Effect on rate ratios of adjusting for age and removal from the risk set for estimated incarceration periods, all crimes**

|  |  | Not accounting for estimated incarceration | | | Accounting for estimated incarceration | | |
| --- | --- | --- | --- | --- | --- | --- | --- |
|  | Number of offences | Person years (rate) | RR | aRR | Person years (rate) | RR | aRR |
| Men |  |  |  |  |  |  |  |
| Negative | 194,195 | 145,286 (1.34) | Ref | Ref | 140,891 (1.38) | Ref | Ref |
| Cocaine only | 39,053 | 32,860 (1.19) | 0.89 [0.88, 0.90] | 0.92 [0.92, 0.93] | 31,625 (1.23) | 0.90 [0.89, 0.91] | 0.93 [0.92, 0.94] |
| Opiate only | 26,990 | 14,092 (1.92) | 1.43 [1.41, 1.45] | 1.60 [1.58, 1.62] | 13,183 (2.05) | 1.49 [1.47, 1.50] | 1.66 [1.64, 1.68] |
| Opiate and cocaine | 45,976 | 22,908 (2.01) | 1.50[1.49, 1.52] | 1.70 [1.68, 1.71] | 21,245 (2.16) | 1.57 [1.56, 1.59] | 1.77 [1.75, 1.79] |
| Women |  |  |  |  |  |  |  |
| Negative | 30,145 | 47,894 (0.63) | Ref | Ref | 47,701 (0.63) | Ref | Ref |
| Cocaine only | 5,827 | 5,418 (1.08) | 1.71 [1.66, 1.76] | 1.67 [1.63, 1.72] | 5,344 (1.09) | 1.73 [1.68, 1.77] | 1.68 [1.64, 1.74] |
| Opiate only | 6,715 | 3,992 (1.68) | 2.67 [2.60, 2.74] | 2.68 [2.61, 2.75] | 3,904 (1.72) | 2.72 [2.65, 2.79] | 2.73 [2.66, 2.80] |
| Opiate and cocaine | 15,944 | 7,400 (2.15) | 3.42 [3.36, 3.49] | 3.37 [3.31, 3.44] | 7,179 (2.22) | 3.51 [3.45, 3.58] | 3.46 [3.39, 3.53] |

Rate = number of offences per person year; RR = rate ratio; aRR = rate ratio adjusted for age

**Supplementary Material C.1 Results from Poisson regression - effect of covariate age at drug test, adjusted for DTR result**

|  | Age at drug test | | | | | |
| --- | --- | --- | --- | --- | --- | --- |
|  | 18-19 | 20-24 | 25-29 | 30-39 | 40-49 | >50 |
| **Males** |  |  |  |  |  |  |
| All | 1.50 [1.48, 1.52] | 1.09 [1.08, 1.10] | Ref | 0.94 [0.93, 0.95] | 0.81 [0.80, 0.82] | 0.62 [0.60, 0.63] |
| Acquisitive crimes | 1.27 [1.25, 1.30] | 0.90 [0.89, 0.92] | Ref | 0.98 [0.96, 1.00] | 0.86 [0.84, 0.88] | 0.70 [0.67, 0.72] |
| Serious acquisitive | 2.57 [2.48, 2.67] | 1.08 [1.04, 1.13] | Ref | 0.85 [0.82, 0.88] | 0.52 [0.49, 0.55] | 0.27 [0.24, 0.31] |
| Non-serious acquisitive | 0.87 [0.84, 0.89] | 0.85 [0.83, 0.87] | Ref | 1.02 [1.00, 1.04] | 0.97 [0.95, 0.99] | 0.83 [0.80, 0.86] |
| Non-acquisitive | 1.63 [1.61, 1.66] | 1.19 [1.17, 1.21] | Ref | 0.92 [0.90, 0.93] | 0.79 [0.77, 0.80] | 0.58 [0.56, 0.60] |
| Violence atp | 1.68 [1.62, 1.75] | 1.23 [1.18, 1.28] | Ref | 0.95 [0.92, 0.99] | 0.80 [0.76, 0.84] | 0.46 [0.42, 0.51] |
| Sexual | 0.96 [0.80, 1.15] | 0.88 [0.74, 1.04] | Ref | 1.14 [0.97, 1.35] | 1.59 [1.33, 1.90] | 1.79 [1.43, 2.25] |
| Burglary | 1.79 [1.70, 1.88] | 0.94 [0.89, 0.99] | Ref | 0.96 [0.91, 1.01] | 0.67 [0.63, 0.71] | 0.33 [0.28, 0.38] |
| Robbery | 5.43 [4.77, 6.18] | 1.52 [1.32, 1.75] | Ref | 0.63 [0.53, 0.75] | 0.45 [0.36, 0.57] | 0.18 [0.10, 0.32] |
| Theft from person | 2.07 [1.78, 2.40] | 1.30 [1.12, 1.51] | Ref | 1.14 [0.99, 1.33] | 1.09 [0.91, 1.31] | 0.68 [0.49, 0.95] |
| Theft of vehicle | 5.36 [4.94, 5.81] | 1.83 [1.67, 1.99] | Ref | 0.71 [0.64, 0.78] | 0.35 [0.30, 0.41] | 0.32 [0.24, 0.42] |
| Stealing from vehicle | 1.60 [1.47, 1.75] | 0.80 [0.73, 0.87] | Ref | 0.70 [0.64, 0.76] | 0.27 [0.23, 0.32] | 0.11 [0.07, 0.17] |
| Shoplifting | 0.68 [0.65, 0.71] | 0.73 [0.71, 0.76] | Ref | 1.04 [1.01, 1.07] | 0.99 [0.95, 1.02] | 0.88 [0.83, 0.93] |
| Other theft | 1.18 [1.13, 1.23] | 0.94 [0.90, 0.98] | Ref | 1.00 [0.96, 1.05] | 0.93 [0.89, 0.98] | 0.87 [0.80, 0.94] |
| Fraud and forgery | 0.52 [0.48, 0.55] | 0.77 [0.73, 0.81] | Ref | 1.05 [1.00, 1.10] | 0.99 [0.93, 1.05] | 0.73 [0.67, 0.81] |
| Criminal damage | 2.28 [2.16, 2.39] | 1.34 [1.28, 1.41] | Ref | 0.83 [0.79, 0.88] | 0.61 [0.56, 0.65] | 0.38 [0.33, 0.44] |
| Drug (misuse) | 1.54 [1.47, 1.62] | 1.20 [1.15, 1.26] | Ref | 0.86 [0.82, 0.90] | 0.66 [0.62, 0.71] | 0.41 [0.36, 0.46] |
| Drug (supply) | 1.28 [1.19, 1.38] | 1.18 [1.10, 1.27] | Ref | 0.90 [0.84, 0.96] | 0.92 [0.84, 1.00] | 0.69 [0.60, 0.80] |
| Other indictable | 2.90 [2.72, 3.09] | 1.40 [1.31, 1.50] | Ref | 0.93 [0.87, 1.00] | 1.01 [0.93, 1.10] | 1.52 [1.37, 1.69] |
| Summary | 1.38 [1.35, 1.42] | 1.17 [1.14, 1.19] | Ref | 0.90 [0.88, 0.92] | 0.83 [0.80, 0.85] | 0.65 [0.62, 0.69] |
| Breach | 1.60 [1.56, 1.64] | 1.15 [1.12, 1.18] | Ref | 0.93 [0.91, 0.96] | 0.73 [0.71, 0.75] | 0.45 [0.42, 0.48] |
| **Females** |  |  |  |  |  |  |
| All | 1.04 [1.01, 1.07] | 0.93 [0.91, 0.95] | Ref | 0.93 [0.91, 0.95] | 0.73 [0.71, 0.75] | 0.50 [0.47, 0.52] |
| Acquisitive crimes | 0.87 [0.83, 0.90] | 0.91 [0.88, 0.94] | Ref | 0.95 [0.93, 0.99] | 0.84 [0.80, 0.87] | 0.69 [0.65, 0.74] |
| Serious acquisitive | 2.69 [2.30, 3.15] | 1.23 [1.06, 1.44] | Ref | 0.90 [0.77, 1.05] | 0.50 [0.40, 0.64] | 0.37 [0.24, 0.57] |
| Non-serious acquisitive | 0.79 [0.76, 0.83] | 0.90 [0.87, 0.93] | Ref | 0.96 [0.93, 0.99] | 0.85 [0.82, 0.89] | 0.70 [0.66, 0.75] |
| Non-acquisitive | 1.28 [1.23, 1.33] | 0.95 [0.91, 0.98] | Ref | 0.90 [0.87, 0.93] | 0.61 [0.58, 0.64] | 0.27 [0.24, 0.30] |
| Violence atp | 2.17 [1.96, 2.39] | 1.15 [1.04, 1.26] | Ref | 1.00 [0.91, 1.11] | 0.64 [0.56, 0.74] | 0.18 [0.12, 0.25] |
| Prostitution | 0.32 [0.22, 0.46] | 0.60 [0.51, 0.72] | Ref | 0.94 [0.82, 1.08] | 0.57 [0.46, 0.70] | 0.82 [0.57, 1.17] |
| Burglary | 1.84 [1.50, 2.25] | 1.03 [0.85, 1.25] | Ref | 0.80 [0.66, 0.96] | 0.52 [0.40, 0.69] | 0.47 [0.29, 0.75] |
| Robbery | 8.32 [4.74, 14.6] | 2.67 [1.49, 4.78] | Ref | 1.49 [0.80, 2.76] | 0.69 [0.28, 1.70] | 0.28 [0.04, 2.11] |
| Theft from person | 1.49 [1.18, 1.88] | 1.05 [0.85, 1.30] | Ref | 0.93 [0.76, 1.14] | 1.02 [0.80, 1.29] | 0.66 [0.42, 1.02] |
| Theft of vehicle | 4.01 [2.80, 5.74] | 1.66 [1.15, 2.39] | Ref | 1.19 [0.81, 1.73] | 0.51 [0.28, 0.92] | 0.10 [0.01, 0.70] |
| Stealing from vehicle | 3.59 [1.96, 6.57] | 1.22 [0.66, 2.28] | Ref | 0.89 [0.48, 1.66] | 0.18 [0.04, 0.77] | 0.29 [0.04, 2.18] |
| Shoplifting | 0.91 [0.85, 0.97] | 0.83 [0.79, 0.88] | Ref | 0.90 [0.86, 0.94] | 0.71 [0.66, 0.75] | 0.52 [0.47, 0.59] |
| Other theft | 0.91 [0.83, 0.99] | 1.06 [0.99, 1.14] | Ref | 0.98 [0.91, 1.05] | 1.02 [0.94, 1.11] | 0.87 [0.77, 0.99] |
| Fraud and forgery | 0.54 [0.48, 0.60] | 0.94 [0.87, 1.01] | Ref | 1.08 [1.00, 1.16] | 1.07 [0.98, 1.16] | 0.94 [0.83, 1.06] |
| Criminal damage | 2.17 [1.85, 2.55] | 1.20 [1.02, 1.40] | Ref | 0.86 [0.72, 1.01] | 0.60 [0.48, 0.76] | 0.35 [0.23, 0.53] |
| Drug (misuse) | 0.76 [0.63, 0.92] | 0.94 [0.82, 1.07] | Ref | 0.94 [0.83, 1.07] | 0.59 [0.49, 0.71] | 0.26 [0.17, 0.40] |
| Drug (supply) | 0.80 [0.62, 1.04] | 0.89 [0.74, 1.08] | Ref | 1.19 [1.00, 1.41] | 0.91 [0.73, 1.14] | 0.29 [0.17, 0.52] |
| Other indictable | 1.17 [0.96, 1.42] | 0.84 [0.71, 0.99] | Ref | 0.72 [0.61, 0.84] | 0.70 [0.57, 0.86] | 0.47 [0.31, 0.69] |
| Summary | 1.11 [1.02, 1.21] | 0.85 [0.79, 0.92] | Ref | 0.85 [0.79, 0.92] | 0.65 [0.59, 0.72] | 0.38 [0.31, 0.45] |
| Breach | 1.01 [0.95, 1.08] | 0.94 [0.89, 0.98] | Ref | 0.91 [0.87, 0.95] | 0.58 [0.54, 0.62] | 0.21 [0.18, 0.25] |

aRR = rate ratio adjusting for drug test result; Ref = reference category; atp = against the person

Numbers in square brackets are 95% confidence intervals

**Supplementary Material C.2 Count of offences by DTR result and crime category**

|  | **Men** | | | | **Women** | | | |
| --- | --- | --- | --- | --- | --- | --- | --- | --- |
| **Crime category** | **Dually negative** | **Cocaine only** | **Opiate only** | **Opiate and cocaine** | **Dually negative** | **Cocaine only** | **Opiate only** | **Opiate and cocaine** |
| All offences | 194195 | 39053 | 26990 | 45976 | 30145 | 5827 | 6715 | 15944 |
| Acquisitive offences | 64167 | 11765 | 11249 | 20433 | 16667 | 2844 | 3419 | 8332 |
| Serious acquisitive offences | 18712 | 3470 | 2359 | 4420 | 813 | 177 | 154 | 369 |
| Non-serious acquisitive offences | 45455 | 8295 | 8890 | 16013 | 15854 | 2667 | 3265 | 7963 |
| Non acquisitive offences | 120592 | 24667 | 14339 | 23046 | 12803 | 2765 | 3040 | 7070 |
| Burglary | 9134 | 1788 | 1431 | 2716 | 439 | 112 | 111 | 259 |
| Theft of vehicle | 5014 | 828 | 328 | 563 | 203 | 40 | 24 | 53 |
| Theft from vehicle | 2522 | 592 | 488 | 940 | 48 | 9 | 10 | 29 |
| Robbery | 2042 | 262 | 112 | 201 | 123 | 16 | 9 | 28 |
| Shoplifting | 15570 | 3611 | 5674 | 10711 | 5229 | 1317 | 2044 | 5022 |
| Other theft and handling | 14803 | 2091 | 1554 | 2618 | 4886 | 475 | 499 | 1005 |
| Fraud and forgery | 9391 | 1176 | 743 | 1171 | 4669 | 450 | 421 | 749 |
| Drug supply offences | 4466 | 1206 | 776 | 1203 | 453 | 111 | 137 | 267 |
| Theft from person | 1225 | 211 | 143 | 310 | 458 | 82 | 54 | 232 |
| Prostitution | - | - | - | - | 159 | 232 | 110 | 688 |
| Other summary offences | 46136 | 9614 | 4787 | 7563 | 3597 | 635 | 533 | 1162 |
| Breach | 36640 | 7865 | 6637 | 11088 | 4923 | 1471 | 1956 | 4846 |
| Violence against the person | 17913 | 3622 | 1461 | 2241 | 2622 | 396 | 368 | 548 |
| Criminal damage | 11525 | 1975 | 702 | 968 | 1088 | 121 | 97 | 140 |
| Other indictable offences | 7211 | 1429 | 701 | 1087 | 573 | 142 | 86 | 374 |
| Sexual | 1167 | 162 | 51 | 99 | - | - | - | - |

**Supplementary Material D Separate analysis on those who were arrested for a trigger offence and those who were not.**

|  | Those arrested for a trigger offence | | | Those not arrested for a trigger offence | | |
| --- | --- | --- | --- | --- | --- | --- |
|  | Rate | RR | aRR | Rate | RR | aRR |
| Males |  |  |  |  |  |  |
| Negative | 1.39 | Ref | Ref | 1.24 | Ref | Ref |
| Cocaine only | 1.22 | 0.88 [0.87, 0.89] | 0.91 [0.90, 0.92] | 1.35 | 1.09 [1.05, 1.13] | 1.11 [1.07, 1.16] |
| Opiate only | 2.06 | 1.48 [1.47, 1.50] | 1.66 [1.64, 1.68] | 1.81 | 1.46 [1.39, 1.54] | 1.62 [1.53, 1.71] |
| Opiate and cocaine | 2.16 | 1.56 [1.54, 1.57] | 1.76 [1.74, 1.78] | 2.17 | 1.75 [1.68, 1.82] | 1.96 [1.88, 2.04] |
| Females |  |  |  |  |  |  |
| Negative | 0.63 | Ref | Ref | 0.75 | Ref | Ref |
| Cocaine only | 1.07 | 1.71 [1.66, 1.76] | 1.67 [1.62, 1.72] | 1.26 | 1.68 [1.53, 1.83] | 1.70 [1.56, 1.86] |
| Opiate only | 1.72 | 2.74 [2.67, 2.82] | 2.75 [2.67, 2.82] | 1.78 | 2.36 [2.11, 2.64] | 2.43 [2.17, 2.72] |
| Opiate and cocaine | 2.24 | 3.57 [3.50, 3.64] | 3.51 [3.43, 3.58] | 2.09 | 2.78 [2.60, 2.98] | 2.89 [2.69, 3.10] |

Rate = number of offences per person year; RR = rate ratio; aRR = rate ratio adjusted for age
